# Apoptosis induced by parasitic diseases

**DOI:** 10.1186/1756-3305-3-106

**Published:** 2010-11-17

**Authors:** Anne-Lise Bienvenu, Elena Gonzalez-Rey, Stephane Picot

**Affiliations:** 1 Malaria Research Unit, University Lyon 1, 8 avenue Rockefeller, 69373 Lyon cedex 08, France; 2 Institute of Parasitology and Biomedicine, CSIC, PT Ciencias de La Salud, 18100 Granada, Spain

## Abstract

Fatalities caused by parasitic infections often occur as a result of tissue injury that results from a form of host-cell death known as apoptosis. However, instead of being pathogenic, parasite-induced apoptosis may facilitate host survival. Consequently, it is of utmost importance to decipher and understand the process and the role of apoptosis induced or controlled by parasites in humans. Despite this, few studies provide definitive knowledge of parasite-induced host-cell apoptosis. Here, the focus is on a consideration of host-cell apoptosis as either a pathogenic feature or as a factor enabling parasite survival and development.

Cell death by apoptotic-like mechanisms could be described as a ride to death with a return ticket, as initiation of the pathway may be reversed, with the potential that it could be manipulated for therapeutic purposes. The management of host-cell apoptosis could thus be an adjunctive factor for parasitic disease treatment. Evidence that the apoptotic process could be reversed by anti-apoptotic drugs has recently been obtained, leading to the possibility of host-cell rescue after injury. An important issue will be to predict the beneficial or deleterious effects of controlling human cell death by apoptotic-like mechanisms during parasitic diseases.

## Apoptosis modulation: harmful or helpful?

Apoptosis is a form of programmed cell death involved in a wide range of adaptive processes, from embryogenesis to stress injury responses. The main benefits of apoptosis occur when an organism is uninfected. However, detrimental effects caused by apoptosis can be triggered by parasitic infection, depending upon the specific host-parasite situation. During their evolution, parasites have developed mechanisms to induce or avoid host cell apoptosis in order to be able to survive and complete their life cycle.

Pathways involved in apoptosis are highly regulated, demonstrating that this mechanism is finely tuned according to the biological environment of the cell. Among the factors involved in that balance in infected organisms, the time of apoptosis (early or late occurrence), the cell type and the type of parasitism (intracellular or not) are the major modulators. For example, early apoptosis of host cells could contribute towards their fight against infection by intracellular parasites; equally, early apoptosis could favour the penetration of the parasite. Late apoptosis of cells of the defence system could be beneficial to the host, clearing excess cells and thereby avoiding the detrimental effects of excessive inflammatory response in the tissue that they would cause (e.g. the deleterious effect of reactive oxygen species or pro-inflammatory cytokines triggered by *Plasmodium *in the liver) [1]. This could also be beneficial for the parasite, by limiting the potential for the host to develop a protective immune response.

A major issue that has not yet been addressed by experimental data is the helpful/harmful ratio obtained with anti-apoptotic treatment during infection. Since apoptosis pathways are common to almost all human cells, it could be speculated that protective adjuvant therapy using anti-apoptotic drug would have a beneficial action on damaged cells but, at the same time, it could also induce deleterious effects on tissues where apoptosis was necessary for cell homeostasis. One other option could be to favour apoptosis of the infected cell. What would be the effect of this treatment on non-infected cells? Treatment devoted to the induction of infected-cell apoptosis, for example to avoid the dissemination of intracellular parasites, could also be responsible for detrimental effects on non-infected cells. Would this increase the aging of normal cells? As yet, there is no evidence for a risk, but these major points should be kept in mind before using a pro-apoptotic treatment.

## When should cell death be called apoptosis?

During the last decade, descriptions of cell death induced by pathogens increased very rapidly. However, cell death is a very complex phenomenon and descriptions were, in many cases, based on methods available in a particular laboratory rather than standardized methods. None of the single morphological, enzymatic or functional aspects of cell death provide a definitive proof that observed cell death should be considered as apoptosis. While a first round of recommendations was published in 2005 [2], a more accurate classification of cell death has been available since late 2008 [3]. It should be mentioned that almost none of the papers reviewed here use the recent classification. A number of cited publications mentioned the use of only one method to explore cell death, and in most cases it is extrapolated from this evidence to be "apoptosis". It should also be kept in mind that cell death could display mixed features and that events observed in a tissue collected *post-mortem *or a cell culture *in vitro *are probably not perfectly representative of the *in vivo *physiology. The inflation of terms used to describe cell death is not related to the same inflation of knowledge on pathological mechanisms. Since it was unrealistic to analyze the published literature with regard to the recent classification system, author's definitions of apoptosis as the mode of cell death that they have observed should be used with caution.

Here, apoptosis of host cells during parasitic disease and its potential association with clinical features will be reviewed. Apoptosis of host immune cells will not be considered in this review.

### Apoptosis of Endothelial cells

Severe malaria is responsible for an unacceptable mortality rate, despite efficient anti-malaria treatment. One explanation for this case fatality rate is host-cell apoptosis induced by the parasite, which could lead to irreversible tissue injuries. Endothelial cells are key players in the pathophysiology of severe disease. Parasitized red blood cells (PRBC) circulating within microvasculature induce a pro-inflammatory cascade leading to activation of endothelial cells. One of the early phenomena occurring in that process is the over-expression of ICAM-1 receptors by endothelial cells (EC) stimulated by the parasite [4]. This leads to parasitized red blood cell (PRBC) adhesion to endothelial cells. Concomitant expression of iNOS results in PRBC sequestration and finally caspase 8 and 9 dependent apoptosis in endothelial cells (EC) [5]. This endothelial cell apoptosis leads to blood-brain-barrier disruption and contributes to the development of cerebral malaria [6]. Authors compared human brains collected *post mortem *after cerebral malaria (CM) with brains collected after death from non-malarial causes [7]. Using immunochemistry methods, they observed a generalized reduction of cell junction proteins (occludin, vinculin, ZO-1) in the brain of patients who died of CM. More recently, it was demonstrated that PRBC adherence to host endothelium is not a prerequisite for inducing apoptosis as sera from malaria patients induced endothelial apoptosis [8]. In addition, *Plasmodium falciparum *field isolates from patients presenting neurological signs can induce EC apoptosis [9] and a soluble apoptotic factor from PRBC could have apoptogenic effects on human brain endothelial and neuroglial cells [10]. Finally, the Rho kinase pathway could be involved in pathogenic interactions between endothelium and *P. falciparum *during CM [11]. Indeed, fasudil, a Rho kinase inhibitor, restores endothelial barrier integrity after exposure of human lung endothelial cells to PRBCs *in vitro*.

While the deleterious effect of brain perivascular oedema and ring haemorrhages are well known, deciphering the exact role that *Plasmodium*-induced endothelial cell-apoptosis plays in CM could help in understanding discrepancies amongst patients living in the same conditions but presenting either severe or non-severe malaria. It may provide a new understanding on the difference in the severity of disease induced by *P. falciparum *compared to *P. vivax, P. ovale *or *P. malariae*. It will also provide a good opportunity for assessment of treatment devoted to the protection of that non specific, but highly regulated, mechanism of endothelial cell death.

Transmitted by the bite of tsetse flies, human african trypanosomiasis (HAT), also known as sleeping sickness, develops after *Trypanosoma brucei *has invaded the central nervous system. This infection causes neuronal demyelination and apoptosis after blood-brain barrier rupture. The damage to brain microvascular endothelial cells is driven by a parasite-derived factor which is released into the bloodstream and cerebrospinal fluid (CSF) during peak parasitaemia and leads to apoptosis in cells of the cerebellum and brain-stem, as demonstrated by terminal deoxynucleotidyl transferase dUTP nick end labelling (TUNEL). The trypanosoma-derived factor was partially characterized and a synthetic peptide made which induces similar apoptosis *in vitro *[12]. Moreover, CSF collected from patients with HAT induced apoptosis in endothelial and microglial cells *in vitro *and contains sFasL and anti-Fas antibodies at higher levels than controls, providing candidate markers of blood-brain barrier disruption [13].

Acute myocarditis is a frequent complication of Chagas disease, caused by *Trypanosoma cruzi*. Cardiac microvasculature alterations, with the formation of microthrombi, were demonstrated in acute cases [14]. However, while apoptosis of endothelial cells occurs, it seems to be infrequent and not associated with invasion of endothelial cells by the parasite. Mediators of inflammation, including reactive oxygen species, are suspected to be involved in this phenomenon [15].

### Apoptosis of cardiomyocytes

Apoptosis of cardiomyocytes was previously reported during ischemia-reperfusion *in vivo*, myocardial infarction [16] and many acute and chronic diseases affecting the heart. Few parasitic diseases lead to cardiac dysfunction and the most relevant is certainly Chagas disease.

Infection by *T. cruzi *leads to multi-organ disease and acute myocarditis is prominent. Cardiac muscle cells are invaded by the parasite, allowing parasite differentiation and multiplication. The occurrence of cardiomyocyte-apoptosis has been demonstrated experimentally.

Using a canine model of acute chagasic myocarditis, apoptosis (identified by TUNEL) was found in non-infected *T. cruzi *cardiomyocytes in close proximity to mononuclear inflammatory cells and was probably caused by the release of toxic mediator of inflammation [14,15]. In these investigations, infected cardiomyocytes did not show evidence for apoptosis as measured by TUNEL, but contradictory data were obtained later [17]. Authors of the latter study speculated that apoptosis of cardiomyocytes is not induced by a mediator of inflammation but by previously described transialidases produced by the parasites. Since there are several lines of evidence that *T. cruzi *is able to up- or down-regulate apoptosis of fibroblasts and macrophages, these findings provide more support to the hypothesis that heart injury during Chagas disease is not only caused by direct invasion of cardiomyocytes by parasites, but by a more complex host-parasite relationship. These authors also related the role of apoptosis in the pathogenesis of the disease to the clone of parasite and their different abilities to invade and proliferate within host cells [17].

The degree to which apoptosis of myocytes occurred during the disease is variable in size and distribution. Using heart samples from chagasic patients, cardiomyocyte-apoptosis was shown and associated with heart failure [18]. It was demonstrated later that an increase in the level of Fas-FasL regulates the extent of cardiac inflammation, cardiomyocytes destruction and heart failure in Chagas disease patients [19,20].

Chagas disease is more chronic than severe malaria, and host cells apoptosis seems to play a different role in the pathology. While infected cells may be partly protected from death, an apoptotic signal is sent to neighbouring cells, extending the area of cardiac cell death. Our knowledge of the exact mechanisms of that process is far from complete, but it could be speculated that, with further understanding, the inhibition of this apoptosis could be a cornerstone in the prevention of heart failures for chagasic patients.

Severe malaria is associated with PRBC sequestration in brain vessels, but this phenomenon is also reported in other organs, including lung, kidney and heart [21,22]. However, the relationship between sequestration and tissue injury is weak in most of these cases. Heart failures have been noticed in very few cases of severe malaria, with no clear relevance of a pro-apoptotic effect caused by the parasite.

More recently, glycosylphosphatidylinositols (GPIs), a class of glycolipids with various functions, was suspected to be involved in cardiomyocyte death [23]. *P. falciparum *GPI (Pf-GPI) failed to induce myocardiocyte-apoptosis *in vitro*. Pf-GPI injected *in vivo *led to the occurrence of apoptotic cells in liver and spleen tissue. Gene expression analysis of Pf-GPI-treated cardiomyocytes showed an up-regulation of both apoptotic genes (*apaf-1, bax*) and a myocardial damage marker (brain natriuretic peptide), while a down-regulation for the anti-apoptotic gene (*bcl-2*) was observed [24].

### Apoptosis of neuronal cells

After brain blood barrier (BBB) disruption caused by endothelial cell-apoptosis, oedema and perivascular haemorrhages are responsible for neuronal cell injury. Axons are the most susceptible to ischemic or toxic damage in different brain areas, and axonal injury detected by elevated CSF levels of tau protein and S-100B (markers of axonal damage) were observed during cerebral malaria in Kenyan children [25]. These authors demonstrated that seizures after admission are a manifestation of brain damage, rather than a cause.

Cognitive deficit is seen during CM and it could be related to astrocytes and microglia cell death. It is suspected that Fas-Fas ligand could be involved in brain-cell apoptosis. In wild type mice, Fas ligand positive cells are localized around the vasculature and particularly in the olfactory bulb. Moreover, Lpr (lymphoproliferation spontaneous mutation) and Gld (generalized lymphoproliferative disease) mice, that have mutations in Fas and Fasl, respectively, are resistant to experimental cerebral malaria despite high parasitaemia levels and brain petechial haemorrhages [26]. This is in contradiction with a previous study reporting that Lpr and Gld mice were susceptible to cerebral malaria [27]. Brains of terminally ill infected mice showed apoptosis of neuronal cells, as detected by TUNEL and caspase 3 antibody binding [28]. Activated caspase 3 detected in brain extracts is correlated with the clinical severity of the disease [29]. Activated caspase 3 was mainly detected in neurons and oligodendrocytes in terminally ill animals, leading authors to suggest that these cells are the most vulnerable to apoptosis.

Clearly, many aspects of the pathogenesis of cerebral malaria are still unknown. It could be speculated that the role of apoptosis was underestimated in the past, and should warrant more attention in the future.

In *T. brucei*-infections, the invasion of the central nervous system by the parasite induces a meningo-encephalitis with BBB disruption and neuronal demyelinisation. Scattered small-cell apoptosis was found in the brain of rats infected with *T. brucei*, as well as degeneration of nerve fibres [30].

In humans, extensive cerebellum and brain-stem apoptosis (detected by TUNEL) was demonstrated, as was injury in the *locus ceruleus*, which is responsible for the modification of sleep and wakefulness characteristic of the disease [13,31]. The parasite's entry into host tissue is mediated by the production of a parasite-derived factor (TAF) which induces endothelial cell-apoptosis and damage to the vascular endothelium. This pathogenic factor, identified by Surface Enhanced Laser Desorption Ionization (SELDI), could be responsible for the destruction of immune cells and apoptosis of brain cells at peak parasitemia [31]. This pro-apoptotic parasitic factor is released in the CSF of patients

Cerebral toxoplasmosis is a severe complication of *Toxoplasma *infection observed in immunosuppressed people and AIDS patients and is associated with a high mortality rate. Few studies have addressed the question of the role of host cell apoptosis in the brain during toxoplasmosis, while more data are available from retina studies. In an experimental model of acquired ocular toxoplasmosis, wild type, lpr and gld mice, all showed a mild to moderate encephalitis with focal necrosis, cerebral vascularitis and meningitidis at day 14, and a moderate to severe disease at day 28 after inoculation [32]. Among the inflammatory cells, TUNEL positive cells were observed in all groups of mice. Expression of Fas/FasL was enhanced in the brain, 28 days after inoculation of wild-type mice with *Toxoplasma gondii*. As expected, this expression was absent in lpr and gld mice. Cerebral toxoplasmosis may be partially explained by Fas/FasL-mediated apoptosis. Other pathways are suspected to be involved in apoptosis, as demonstrated by TUNEL positive cells in the brains of lpr and gld mice.

### Apoptosis of hepatocytes

Infection of hepatocytes after sporozoite transmission to humans by mosquito bites is a crucial step for malaria infection. The relationship between parasites and hepatocytes is complex. It was recently demonstrated that Kupffer cells were TUNEL positive after incubation *in vitro *with sporozoites, suggesting that *P. yoelii *sporozoites caused apoptosis of Kupffer cells [1]. This apoptosis limits the local production of cytokines and the activation of the immune system. However, to survive and develop within hepatocytes, Plasmodium parasites have developed the capability to inhibit host cell apoptosis [33]. Hepatocytes damage induces the release of hepatocyte growth factor [34] mediating the inhibition of the death of infected hepatocytes.

When HepG2 liver cells are infected with *P. berghei*, cells are protected from peroxide injury, whilst non infected HepG2 cells undergo apoptosis [35]. This was confirmed by the demonstration that infected cells containing viable parasites are protected from apoptosis, whereas hepatocytes containing dead parasites are no longer protected from cell death. The levels of Fas expression and caspase 8 activities are the same in infected and uninfected liver cells of mice, but hydroxyl radicals are increased in infected mice [36]. The contribution of oxidative stress to hepatocytes apoptosis has also been demonstrated using melatonin, a non toxic anti-apoptotic and anti-oxidant molecule [37]; melatonin prevents hepatocytes from undergoing apoptosis in mice, as demonstrated by caspase 3 and TUNEL assays.

After parasites have been released from hepatocytes, they invade red blood cells for the erythrocytic multiplication phase. During that stage of the infection, *Plasmodium *degrades huge quantities of haemoglobin to produce heme and haemozoin which are deleterious. Haemozoin can be deposited in the liver, where it is responsible for an oxidative insult [38]. Inside the cytosol, heme is degraded by haem oxygenase 1 (HO-1) which causes the release of iron inside the cell. The existence of high levels of free iron and H_2_O_2 _inside mitochondria favours the generation of highly toxic °OH, oxidation of mitochondrial lipids, release of cytochrome c into the cytosol, and activation of caspase 9/caspase 3 to execute hepatocyte apoptosis. BALB/c mice deficient in haem oxygenase are killed by *Plasmodium chabaudi chabaudi *infection [39], irrespective to the level of parasitaemia. These mice developed hepatic failure but not cerebral malaria or other forms of severe malaria. DBA/2 mice also succumb to *P. c. chabaudi *infection with hepatic failure. Transduction of these mice with a vector expressing HO-1 limits the hepatic injury and avoids the mortality [39]. The same study showed that when exposed to free haem plus TNF, hallmarks of apoptosis are detected in hepatocytes *in vitro*. Data obtained from these different models of malaria demonstrate the high level of complexity of the relationship between *Plasmodium *and hepatocytes, and the need for more research.

The late stage of hepatocytes invasion involves the release of parasites into the blood stream. This release occurs following the rupture of the parasitophorous vacuole membrane, and leads to hepatocytes cell death [40]. This hepatocytes cell death contributes to the formation of merosomes and their budding through the endothelium into the blood vessel.

In malaria infections the liver is consider more as an asymptomatic reservoir of parasites during the early phases of the disease rather than a target for pathogenic mechanisms. Researches dedicated to an understanding of hepatocytes-apoptosis could lead to useful information for designing vaccination programmes against *P. falciparum *sporozoites and for prevention of *Plasmodium vivax *hypnozoites recrudescence.

*Entamoeba histolytica *destroys intestinal cells by the release of proteolytic enzymes and then triggers a host inflammatory response in the liver. This response is believed to contribute to tissue damage and abscess formation. More than a decade ago, authors raised the question of whether parasites-induced necrosis or apoptosis of liver cells occurred as a result. After publication of controversial data [41] a severe, combined immunodeficient mouse model of amoebic liver abscess was used to demonstrate that *E. histolytica *induces TUNEL positive cell death and DNA fragmentation in amoebic liver abscesses [42]. This cell death was not reduced in mice with point mutations in the gld gene resulting in the expression of non-functional Fas ligand, nor in mice lacking TNFR1, demonstrating that TNF alpha and Fas/Fas ligand pathways are not involved.

The role of apoptotic cell death was also documented by the treatment of mice with a caspase inhibitor (Z-VAD-fmk) prior to infection with *E. histolytica *[43]. Amoebic liver abscesses were significantly smaller in mice receiving the caspase inhibitor, with less TUNEL positive hepatocytes and less DNA laddering in the abscesses. However, during a transcriptional analysis of the response of mouse liver to *E. histolytica *infection, 81 apoptosis-related genes exhibited transcriptional changes in amoebic liver abscess, 46 were pro-apoptotic [44]. Increased expression of the gene encoding the pro-apoptosis receptor Fas and genes downstream of Fas signalling, as well as genes linked to the TNF receptor pathways, were detected.

Using engineered parasites (HGL-2 trophozoïtes) presenting a disruption of Gal/GalNAc lectin, authors recently tested the hypothesis that adhesion molecules are linked to liver cell death and the pathophysiology of amoebic abscess. TUNEL analysis of liver cells from infected hamsters showed more TUNEL positive hepatocytes during infection with adherent parasite than with lectin deficient parasite [45]. The decrease in adherence and mobility of the parasite was correlated with a decrease in inflammatory response and local liver cell death.

Biliary tract injury associated with infection by the Asian fluke *Clonorchis sinensis *could lead to cholangiocarcinoma. Cell death involving TUNEL-positive hepatocytes was found in tissue around the central vein or portal areas or rats infected with high parasite loads [46]. Whether this cell death was induced by factors released by the parasite or by inflammatory cells is unknown. The expression of Fas, FasL and caspase 3 mRNA was increased in infected livers. Liver cell death induced by *C. sinensis *may contribute to clinical manifestations in patients.

### Apoptosis in the eye

Toxoplasmosis is a benign disease except during pregnancy and immunosuppression, however, it could be responsible for ocular disease, mostly necrotizing retinitis and retinochoroiditis. It has been hypothesised that apoptosis and Fas/FasL pathways could be involved in ocular toxoplasmosis. To test this, lpr mice and gld mice which have defective Fas and FasL expressions respectively, were used [32,47]. A greater number of TUNEL positive cells were observed in the anterior chamber and in vitreous humour of wild-type mice after intraocular infection compared to lpr and gld deficient mice. Fas and FasL expression was increased in the eyes of infected wild-type mice compared to non infected mice [47].

*Acanthamoeba *keratitis is an infection of the ocular surface that produces pain and ulceration. It involves sequential events including production of pathogenic proteases that degrade membranes and induce cytolysis and apoptosis of cornea cells [48]. After adhesion of trophozoïtes to the corneal epithelial cells, *Acanthamoeba *secretes proteases that enable the amoebae to penetrate corneal tissue: mannose-induced protein 133 (MIP 133) produces contact-independent cytolysis of corneal epithelial cells *in vitro *and facilitates destruction of the corneal epithelium [49]. *Acanthamoeba *can also use plasminogen activators to catalyse the cleavage of host plasminogen to form plasmin. Then, plasmin can activate proteases, such as matrix metalloproteinase (MMP) that can degrade components of the extracellular matrix [50] and this process is involved in the observed corneal ulceration [51].

Malarial retinopathy occurs in 60% of children suffering cerebral malaria, with a high fatality rate [52]. During cerebral malaria in humans, pathological examination of eyes *post-mortem *showed parasites sequestered in the retinal vasculature and haemorrhages that were similar to ring haemorrhages seen in the brain and associated with thrombi, and rupture of the blood-retinal-barrier [53]. Due to these similarities between the retina and the brain injury it may be speculated that apoptosis would also be detected in the eye. Evidence of apoptosis in the retina was provided in an experimental model of cerebral malaria. In retinas of wild-type cerebral-malaria mice, it was shown that microglial cells are TUNEL positive and that astrocytes are caspase-3 positive at day 6 post-infection. In contrast, in lpr and gld deficient mice, no apoptosis was observed in the retina after *Plasmodium *infection.

These observations of ocular damage induced during parasitic diseases, mainly due to toxoplasmosis and malaria infection, could provide evidence for concomitant or future cerebral damage. This could help target more accurately patients who would need more intensive treatments to fight future complications of the diseases.

### Apoptosis in the Skin

The expression of Fas in mice skin tissue was reduced in chronically infected animals. This could explain the progression of the lesion in animals [54]. During *L. major *infection, apoptosis in keratinocytes was mainly observed in the superficial epidermis of active and healing skin samples from patients [55]. Fas expression was increased in the superficial epidermis of active samples whereas, in healing and healthy patients, Fas was not increased. Fas-L expression was mainly found in macrophages and T cells of active samples. Fas-L expression of macrophages and T cells may induce apoptosis and contribute to ulcer formation in Fas-expressing keratinocytes during *L. major *infection.

### Apoptosis of Erythrocytes

Intra-erythrocytic parasite development is dependant on the capability of erythrocytes to tolerate parasite invasion and multiplication as this will contribute to an increase in parasitaemia. Intra-erythrocytic parasites age their host cells. They induce a dramatic acceleration of normal erythrocyte senescence which contributes to the clearance of infected cells. The intra-erythrocytic parasites have the challenge of delaying the execution of erythrocyte apoptosis: *P. falciparum *sequesters calcium, decreases the colloid osmotic pressure of the erythrocytic cytosol and decreases phosphatidylserine exposure [56].

Phosphatidylserine exposure is an "eat me" signal for trophozoite infected erythrocytes leading to their phagocytic clearance thus inhibition of its exposure contributes to partial immune evasion.

When apoptosis of erythrocytes occurs it leads to haemozoin discharge. The direct effect of haemozoin on erythroid precursors has been demonstrated [57]. Haemozoin increases levels of reactive oxygen species and of apoptosis in erythroblasts that are responsible for inhibition of erythroid expansion. Activation of caspase 8, 9 and 3 and mitochondrial potential disruption, were observed in erythroblasts, implicating both extrinsic and intrinsic pathways in the execution of apoptosis. However, macrophages can protect erythroblasts from the inhibitory effects of haemozoin [57]. In the absence of inflammatory mediators and macrophages, accumulation of haemozoin in bone marrow could contribute to the severity of anaemia in children with chronic malarial infection.

### Apoptosis in the bowel

*Cryptosporidium parvum *induces apoptosis in intestinal and biliary epithelial cells in vitro [58]. Apoptosis is induced by Fas and FasL protein expression, leading to caspase activation and cleavage of structural target proteins. *C. parvum *can also prevent apoptosis through increase activity of NF-kB [59].

*Trichinella spiralis *induces apoptosis in the lamina propria of the small intestine and also in striated muscle [60]. Apoptosis in the intestinal cells is independent of the number of *Trichuris muris*. Neutralization of IFN gamma and TNF alpha causes a significant reduction in apoptosis, suggesting proinflammatory cytokines may play a positive role by promoting intestinal epithelial-cell apoptosis that limits infection-induced epithelial hyperplasia [61].

## Apoptosis targeting: a therapeutic opportunity for the future?

It is now clear that almost all parasitic diseases induce host-cell death, leading to tissue damage. It is well known that ischemic injury results in necrotic cell death in tissue, surrounded by apoptosis-like cell death in the area named the "ischemic penumbra". In that area, some lines of evidence were recently provided showing that the apoptotic process could be reversed by anti-apoptotic drugs leading to the opportunity to rescue cells. These observations open new doors for tissue protective therapies that avoid or control these deleterious processes [62].

The idea of tissue protection was first successfully explored during experimental and human ischemic stroke. A double-blind, placebo-controlled, randomized trial was conducted on patients suffering from a stroke and demonstrated that high doses of erythropoietin (Epo) (33 000 IU/day during 3 days) was well tolerated and safe [63]. Systemic administration of exogenous Epo regulates cerebral blood flow, protects endothelial cells against oxidative stress, enhances neurogenesis and angiogenesis and reduces inflammation by inhibiting pro-inflammatory cytokines [62]. These effects are partly due to the inhibition of cellular apoptosis [64]. Interestingly, Epo treatment can be initiated in the first 24 hours after initiation of the lesion [65].

To date, very few studies have been dedicated to these anti-apoptotic interventions during parasitic diseases and some anti-apoptotic approaches tested in experimental parasitic diseases, were unsuccessful. For example, treatment with z-VAD, a pan-caspase inhibitor, and overexpression of Bcl-2 in transgenic mice, failed to reduce mortality in experimental cerebral malaria [66]. However, some antiapoptotic interventions in parasitic disease were proven to be more successful. In a mouse model of amaeobiasis, z-VAD was demonstrated to reduce the mean liver size by 70% at 24 hours compared to the control group [43].

The neuroprotective proof of concept, using high doses of Epo during a stroke, was applied to a parasitic feature, cerebral malaria, a complex disorder with many similarities to neurological stroke [67]. During cerebral malaria, reduced cerebral blood flow is the pathological consequence of microcirculation obstruction by infected red blood cell sequestration [68].

Recently, the first evidence for neuroprotective adjuvant therapy using Epo was provided in a murine model of cerebral malaria: an artesunate - erythropoietin drug combination led to an increase in the global survival rate compared to artesunate monotherapy and to a clinical recovery 24 hours earlier for surviving mice [69,70]. Wiese's study confirmed these results and demonstrated that recombinant erythropoietin increases survival in mice with CM in a dose and time dependent manner [28]. This effect could be mediated by neuronal apoptosis reduction: Epo reduced the mean number of TUNEL+ neurons counted in the sagittal sections from both brain hemispheres. Treatment with Epo was able to decrease significantly IL-1β, TNFα and IFNγ. It could also be speculated that Epo's effect is related to a reduction in the production of pro-inflammatory cytokines.

Another rationale for Epo adjunctive therapy during cerebral malaria was recently provided by the demonstration in African children with human cerebral malaria that high levels of Epo are associated with protection against neurological sequelae [71]. In this context, an open-labelled study, including children presenting with very severe malaria with a Blantyre coma score below 3, was conducted in Mali [72]. The objective was to assess the short-term safety (seven days) of erythropoietin at high doses (1,500 U/kg/day during three days) combined with quinine. 35 patients with unrousable coma were included in the study. None of the expected side effects of erythropoietin were observed during the seven days follow-up. No significant increase in the case fatality rate (7/35 patients) was observed compared with other studies with mortality rates ranging from 16 to 22% in similar endemic areas. These data provide the first evidence of the short-term safety of erythropoietin at high doses combined with quinine. A multicenter study is needed to assess the potential of Epo as an adjunctive therapy to increase survival during cerebral malaria.

Taken together, these demonstrations are leading towards new opportunities for cerebral malaria adjuvant therapy in the near future. However, there is a main drawback related to Epo use in a clinical setting due to its potential for blood mass increase, although this is rarely associated with thrombosis. No evidence of this side effect was reported during Ehrenreich's proof of concept study using 33 000 IU/day of Epo during stroke. This is in accordance with our results obtained in children suffering from cerebral malaria and most of the time admitted to hospital with severe anaemia. In this context, compounds having Epo's neuroprotective properties but without haematological side effects could be an exciting alternative [73].

Other cytoprotective strategies have been tested against parasitic diseases. Glatiramer, an immunomodulatory and potentially neuroprotective drug, was shown to lead to lower risk for developing cerebral malaria in treated animals. No direct neuroprotective effect such as inhibition of apoptosis was found, but lower interferon gamma levels were demonstrated in treated animals [74]. This effect could be related to glatiramer-increased anti-inflammatory type II monocytes and glatiramer regulatory T cells promotion, as demonstrated in a model of multiple sclerosis [75]. Fasudil is a Rho kinase inhibitor known to improve endothelial function through the eNOS anti-apoptotic pathway: this drug has a potential for restoration of endothelial barrier integrity after human lung endothelial cell exposure to PRBCs *in vitro *[11]. z-VAD, a caspase inhibitor, was successfully tested in a Chagas model: this drug led to prevention of lymphocytes apoptosis and reduction of parasitemia through type 1 immune response promotion [76].

From these studies, we can clearly identify promising drugs that could be candidates for anti-apoptotic adjuvant treatment in parasitic diseases. Even if these models are not reproducing all the features of human infections, they can predict most of the drug's positive and negative effects. Thus, evidence obtained from animals could open new areas of translational research, working from experimental evidence to clinical settings. Some of these candidates are registered drugs used for many years in humans. In those cases, translation from experimental data to a clinical setting can be accelerated. Despite the availability of safety profiles for these registered drugs, unexpected side effects need to be carefully monitored as they would be for a known drug in a new disease setting.

There is a need to explore these anti-apoptotic adjuvant treatments in parasitic diseases. These new therapeutic opportunities could contribute to the reduction of the unacceptable high mortality rates associated with these diseases.

## Competing interests

The authors declare that they have no competing interests.

## Authors' contributions

All authors contributed equally to the draft of the manuscript. All authors read and approved the final manuscript.
